# Variation and heritability of retinal cone ratios in a free‐ranging population of rhesus macaques

**DOI:** 10.1111/evo.14552

**Published:** 2022-07-19

**Authors:** Rachel A. Munds, Eve B. Cooper, Mareike C. Janiak, Linh Gia Lam, Alex R. DeCasien, Samuel Bauman Surratt, Michael J. Montague, Melween I. Martinez, Cayo Biobank Research Unit, Shoji Kawamura, James P. Higham, Amanda D. Melin

**Affiliations:** ^1^ Department of Anthropology and Archaeology University of Calgary Calgary AB T2N 1N4 Canada; ^2^ Department of Anthropology New York University New York New York 10003; ^3^ New York Consortium in Evolutionary Primatology New York New York 10460; ^4^ School of Science, Engineering and Environment University of Salford Salford M5 4NT United Kingdom; ^5^ Section on Developmental Neurogenomics National Institute of Mental Health Bethesda Maryland 20892; ^6^ Caribbean Primate Research Center University of Puerto Rico San Juan Puerto Rico 00936; ^7^ Department of Neuroscience University of Pennsylvania Philadelphia Pennsylvania 19104; ^8^ Department of Integrated Biosciences University of Tokyo Kashiwa 277‐8562 Japan; ^9^ Department of Medical Genetics University of Calgary Calgary AB T2N 1N4 Canada; ^10^ Alberta Children's Hospital Research Institute University of Calgary Calgary AB T2N 1N4 Canada

**Keywords:** Catarrhine, color vision, droplet digital PCR, gene expression, heritability, opsin

## Abstract

A defining feature of catarrhine primates is uniform trichromacy—the ability to distinguish red (long; L), green (medium; M), and blue (short; S) wavelengths of light. Although the tuning of photoreceptors is conserved, the ratio of L:M cones in the retina is variable within and between species, with human cone ratios differing from other catarrhines. Yet, the sources and structure of variation in cone ratios are poorly understood, precluding a broader understanding of color vision variability. Here, we report a large‐scale study of a pedigreed population of rhesus macaques (*Macaca mulatta*). We collected foveal RNA and analyzed opsin gene expression using cDNA and estimated additive genetic variance of cone ratios. The average L:M ratio and standard error was 1.03:1 ± 0.02. There was no age effect, and genetic contribution to variation was negligible. We found marginal sex effects with females having larger ratios than males. S cone ratios (0.143:1 ± 0.002) had significant genetic variance with a heritability estimate of 43% but did not differ between sexes or age groups. Our results contextualize the derived human condition of L‐cone dominance and provide new information about the heritability of cone ratios and variation in primate color vision.

One of the defining features of catarrhines (African and Asian monkeys and apes, including humans) is the evolution of uniform trichromacy—red, green, and blue color vision that is exhibited by almost all individuals of both sexes. Trichromatic color vision is thought to have had major impacts throughout primate and human evolution. The ability to distinguish reds, oranges, and yellows against green backgrounds, an ability that dichromatic mammals do not have, is likely to have opened up a range of important new dietary niches by allowing the ready detection of ripening fruits, developing new leaves, and blossoming flowers from considerable distances in the forest (Mollon 1989; Dominy and Lucas 2001; Valenta and Melin 2012; Hogan et al. 2018). It also is likely to have had significant impacts on the ability of human ancestors to detect potential predators, including felids and brightly colored venomous snakes (Isbell 2009; Pessoa et al. 2014). The evolution of uniform trichromacy has also been associated with an explosion of reddish sociosexual signals, for which the clade is renowned, from the nose of the male mandrill, the chest patch of the gelada, and the red faces of several macaque species, to the red genitals, hindquarters, and sexual swellings of many catarrhine species (Bradley and Mundy 2008; Dixson 2012; Moreira et al. 2019; Winters et al. 2019). Overall, the impact of trichromacy on diet, predator detection, and social signaling indicates that the ability to easily distinguish reds from greens, and between shades of reddish colors, has been highly important during catarrhine evolution.

Placental mammals are ancestrally dichromatic and, as a consequence, red‐green colorblind. The evolution of routine trichromacy, via a gene duplication on the X‐chromosome, resulted in the emergence of distinct long‐wave (L) and medium‐wave (M) sensitive cone opsin genes (*OPN1LW* and *OPN1MW*, respectively). In humans, the M photoreceptor is maximally sensitive around 530 nm, and the L photoreceptor around 560 nm, and these sensitivities are highly conserved across catarrhines (Jacobs and Deegan 1999). This strong conservation might point to evolutionary constraints acting on catarrhine color vision and cone sensitivities. However, the relative ratio of expression of the L to M cones is different in humans (averaging 2:1) from other catarrhines—including macaques and chimpanzees—(averaging 1:1) (Mollon and Bowmaker 1992; Jacobs et al. 1996; Dobkins et al. 2000). Functional consequences of cone ratio variation include differences in color discrimination thresholds between humans and nonhuman primates (Jacobs and Deegan 1997; Gagin et al. 2014; Lindbloom‐Brown et al. 2014). Interestingly, human and macaque L:M cone ratios also vary intraspecifically—by as much as 30‐fold in humans (Dobkins et al. 2000; Carroll et al. 2002). This raises important questions: Why are L and M cone spectral sensitivities so conserved across catarrhines, whereas the ratio of expression of those cones in the retina is different between humans and other species? It has been hypothesized that human cone ratios are L dominated as an acuity adaptation that has come at some cost to chromatic sensitivity (Gagin et al. 2014). Demonstrating that cone ratios vary and assessing the sources of variation (genetic vs. environmental) are requisites for assessing whether such variation might be under selection. However, these questions have proven extremely hard to address given that large samples are needed from pedigreed populations, and that the required sampling is invasive and destructive.

In addition to L and M cones, S cones, which in humans and other catarrhine primates are sensitive to blue wavelengths of light, are required for trichromatic vision with their signal being compared against a combined L:M signal to allow blue‐yellow color vision (Ripamonti et al. 2009; Schmidt et al. 2016). Most literature addressing primate trichromatic vision has focused on L and M cones given their evolutionary novelty among mammals (Jacobs and Williams 2006; Neitz et al. 2006; Schmidt et al. 2016; Brainard 2019). However, S cones, encoded by the autosomal *OPN1SW* opsin gene, also play an essential role in chromatic vision. S cones are typically expressed in much lower densities than L and M cones, especially in the fovea, which is responsible for highly acute, center‐field vision (Moritz et al. 2014; Brainard 2015). However, the extent of variation of S cones in diurnal monkey retinas is largely unknown, and it is unclear what the sources of any variation might be. More generally, understanding the ratios of retinal cone classes to include S cones is also important given the critical role of these ratios in perceptual models of primate color vision (Ripamonti et al. 2009; Schmidt et al. 2016; Brainard 2019).

A complicating factor in the study of retinal cone expression is the effects of age and sex, about which little is known. Unlike some vertebrates, mammalian retinas do not regenerate or undergo metamorphosis post birth, such that changes in L:M and S: (L+M) cone ratios with age are not predicted (Aboelnour et al. 2017; Kam et al. 2019). However, if an age effect were to be consistently observed, this might indicate disproportionate die‐off of certain cone types during aging. There is evidence for impacts of other factors on cone ratios, including myopia (Hagen et al. 2019), indicating a need to control for as much variation as possible. Moreover, given the location of the L and M loci on the X‐chromosome, and ensuing sex‐linked nature of color vision genotypes, sex might also feasibly impact the ratio of L:M cones. For example, the hemizygous genotype of males has phenotypic consequences (i.e., expression of only L or M cones) in the presence of opsin gene deletion events. Additionally, males are more likely to have color vision anomalies due to loss of opsin genes during unequal crossover events (Sharpe et al. 1999; Simunovic 2010). Females are largely sheltered from such effects by having two copies of each the *OPN1LW* and *OPN1MW* (Sharpe et al. 1999; Wong 2011). Furthermore, male mammals tend to exhibit relatively more variability across morphological traits (e.g., Zajitschek et al. 2020), and sex differences in the variability of cone ratios may be present. Overall, the impacts of age and sex on cone ratio variation are poorly known but potentially important in the context of visual disorders.

Here, we document for the first time the extent of variation in S, M, and L retinal cones in a large free‐ranging primate population and investigate genetic versus environmental (i.e., nongenetic) sources of variation in the trait. We examine the variation and heritability of (1) L:M cone ratios, and (2) S:(L+M) cone ratios in rhesus macaques (*Macaca mulatta*) from Cayo Santiago, using RNA extracted from retinal tissue biobanks. Rhesus macaques are a premier biomedical model for human health research, including studies of visual system evolution and function (Horwitz 2015). Accordingly, ocular tissues derived from the Cayo Santiago population are uniquely positioned for this research due to an extensive pedigree and cross‐sectional sampling across the full life span, and between males and females in a social group living under the same, naturalistic (outdoor, free‐ranging) environmental conditions. We examine patterns of variation in cone ratios and test the alternative hypothesis that intraspecific cone ratio variability is a genetically heritable trait in a free‐ranging primate population, against the null hypothesis that it is not.

## Materials and Methods

### STUDY POPULATION AND TISSUE COLLECTION

The Cayo Santiago population was founded in the late 1930s from a population of 409 individuals captured at various locations in India, and now consists of 1700 individuals, with no evidence of inbreeding (Blomquist 2009), and evidence for genetic outbreeding via dissociative mating (Widdig et al. 2017). Although provisioned with food and water, rhesus macaques on Cayo Santiago live under otherwise naturalistic conditions (Widdig et al. 2017). They are free to interact and move among social groups, and are fully exposed to the environment (daylight, precipitation, temperature fluctuations), such that eye development occurs under natural light conditions. Detailed demographic data are available from 1958, and there is a genetic parentage database that includes animals born from around 1985 onward. The population is managed by the Caribbean Primate Research Center (CPRC), an institute of the University of Puerto Rico.

Retinal foveal tissues were sampled from a tissue biobank that included rhesus macaque samples collected in 2018 and 2019 from the island of Cayo Santiago (Hernandez‐Pacheco et al. 2016). Fovea were collected from 189 rhesus macaques (*Macaca mulatta*), including males (*n* = 82) and females (*n* = 107). The age range was from 2 months to 19 years old, with an average age of 6.2 years. Individuals 15 years of age and older are considered to be “aged” as the life span of rhesus monkeys is three to four times shorter than that of humans (Chiou et al. 2020; Janiak et al. 2021).

Retinal punches (4 mm diameter) that included, and were centered on, the fovea (about 1.5 mm diameter) were performed with a sterile disposable biopsy punch (Sklar surgical instrument, PA Cat #SK96‐1115) under a bright light. All samples were collected during daylight hours, between 8:00 a.m. and 5:30 p.m. The foveal region was chosen because it contains the highest cone density and is responsible for acute color vision. The biopsied tissue was immediately placed in a 2‐mL tube with 500 μL of TRIzol © reagent (Invitrogen) then transferred to a –80° freezer. Samples were shipped frozen to the University of Calgary where they were stored until RNA extraction. All data collection was approved by IACUC (protocol #3380300) of the University of Puerto Rico and the Animal Care Committee (protocol AC19‐0091) of the University of Calgary.

### RNA EXTRACTION

We isolated total RNA from foveal punches preserved in TRIzol solution in a biological safety cabinet using an RNeasy Mini kit (Qiagen Inc. product no. 74106) following manufacturer's protocols, preceded by a chloroform RNA isolation step (Material S1). After this step, we adhered to the Qiagen RNeasy kit protocol, with the addition of a DNase removal step. Our elution amount was 35 μL and included a 10‐min incubation period. RNA concentration was verified using a Qubit 4 Fluorometer following standard protocol (Invitrogen). RNA quality was assessed using a TapeStation (Agilent 2200) on a small subset of the samples (*n* = 8). Our mean (± SE) RNA integrity number (RIN) was 7.5 ± 0.4 with a range of 5.2–8.9, indicating high‐quality starting material.

We transcribed the extracted RNA to cDNA using Bio‐Rad's iScript Rt‐qPCR kit. The RT enzyme in this kit includes a terminal reaction such that the mRNA is only transcribed into cDNA once, resulting in a 1:1 ratio of cDNA from the mRNA transcripts, which is appropriate for gene expression work. A total input of 300 ng, or 100 ng for low‐concentration (<30 ng/μL) samples to avoid exhausting samples, was added to the 20 μL reaction, which also included 4 μL iScript RT Supermix and nuclease‐free water. The reaction was incubated for priming for 5 min at 25°C, reverse transcription for 20 min for 46°C, and RT inactivation for 1 min at 95°C. All cDNA samples were diluted to a 1 ng per 1 μL stock to standardize input across individuals.

### QUANTIFYING RETINAL OPSIN GENE EXPRESSION

Gene expression studies are informative about retinal cone populations in vertebrates, including primates (Deeb et al. 2000; Neitz et al. 2006; Iwanicki et al. 2017; Dong et al. 2019; Wright et al. 2019). Specifically, Deeb et al (2000) have previously demonstrated that the relative expression of mRNA from *OPN1LW* and *OPN1MW* opsin genes correlates well with the relative abundance of the L and M cones in the retinas of macaques. We measured L, M, and S retinal cone concentrations via droplet digital PCR (ddPCR) by quantifying the copies of their respective PCR targets, that is, the mRNA transcribed from *OPN1LW*, *OPN1MW*, and *OPN1SW* opsin genes, respectively (Hindson et al. 2011; Whale et al. 2016). Similar to more traditional gene expression methods, such as quantitative PCR (qPCR), ddPCR uses a Taq polymerase in a standard PCR reaction to amplify a target region either using primers or a primer and probe set (Hindson et al. 2011; Taylor et al. 2017). However, ddPCR is advantageous in that it is more precise, and does not require the additional step of generating a standard curve. Furthermore, ddPCR outperforms qPCR for quantifying targets in low concentrations (Hindson et al. 2011; Taylor et al. 2017). We targeted exon 5 of the *OPN1LW* and *OPN1MW* opsin genes as this exon is the most divergent between the two opsin genes, with a total of six differing base pairs. We designed one set of primers that amplified both the *OPN1LW* and *OPN1MW* opsin genes (Table S2), and we distinguish among them by using distinct probes linked to a fluorescent dye (FAM and HEX for L and M, respectively). This duplex competing assay, where targets share primers and compete for fluorescence, is ideal when looking at highly similar genes (Choudhury et al. 2016; Whale et al. 2016). Additionally, we measured the short wavelength (*OPN1SW*) opsin with a custom S‐probe that was also labeled with HEX dye. S and M droplets were differentiated by varying the amounts of probe added, and thus the overall amount of fluorescence.

To validate our methods and test the efficacy of our primers and probes ahead of experiments with macaque tissues, we designed synthetic cDNA sequences from published rhesus macaque opsin sequences (Choudhury et al. 2016). We ran a series of optimizations using the QX200 Droplet Digital PCR system (Bio‐Rad, USA) that involved single, duplex, and triplex assays to optimize the temperature, and primer, probe, and sample concentration gradients (Table S2). We varied the concentrations of different target opsin genes and confirmed our probes were effective at correctly identifying and distinguishing the opsin genes in the expected concentrations (Fig. S1; Table S3). Furthermore, as both the S probe and M probe shared the same dye (HEX), we optimized the primer and probe concentrations that allowed us to reliably quantify the concentrations of these two genes.

**Figure 1:**
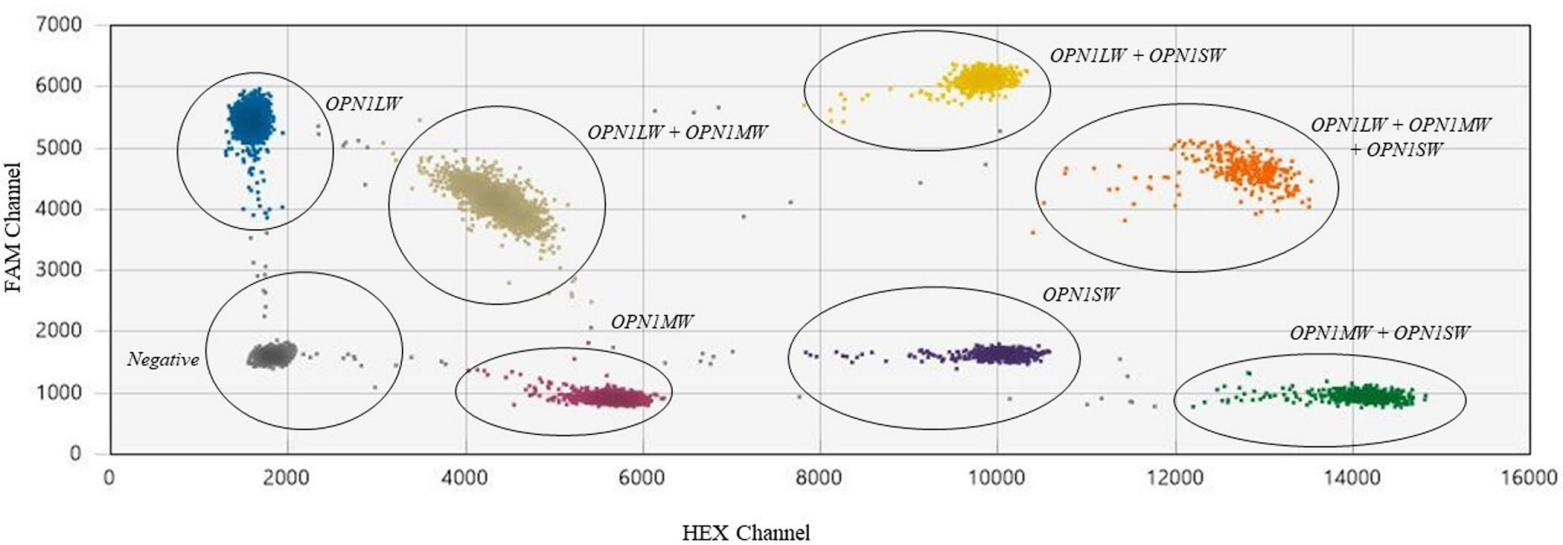
Visualization of the triplex assay measuring opsin gene transcript expression using ddPCR. Eight clusters are identifiable and contain different combinations of pure (e.g., only OPN1LW) and mixed results (e.g., both OPN1MW and OPN1SW present) for the presence of opsin gene expression in each droplet. This ddPCR visualization is a result from one of the macaques in the study.

We quantified opsin gene expression for each macaque retinal tissue sample in triplicate. The ddPCR reaction mixture consisted of 12.5 μL of Supermix for probes (no dUTPs), 1.88 μL of S forward and reverse primers, and 0.53 μL of the S probe. The L and M forward and reverse primers were 2.25 μL with 0.63 μL for both the M and L probes. In addition, 0.47 μL of RNase free water and 2 μL of cDNA were included, resulting in a 25 μL reaction. Once mixed, 22 μL of the reaction was transferred to a DG8 cartridge where 70 μL of droplet generation oil for probes was added. The loaded cartridge was placed in the QX200 Droplet Generator where approximately 15,000 droplets were generated per experiment. In each eight‐well cartridge, one well was left as nontemplate control (water). Droplets were then transferred to a 96‐well plate that was heat‐sealed with a pierceable aluminum foil. We used a C100 Touch Thermal Cycler (Bio‐Rad, US) for amplification (thermal cycle settings in Table S4). The amplified products were then loaded into the QX200 Droplet Reader (Bio‐Rad, USA). Droplets are read sequentially by well, where positive droplets fluoresce and are counted in a two‐color partition, and negative droplets are counted to allow for absolute quantification. This resulted in eight clusters. Three of these clusters contain single‐positive results (only L, M, or S opsin targets). Three of the clusters contain double‐positive results, and one cluster contains droplets positive for all three opsin transcripts. The final cluster contains negative droplets without any amplification (Fig. 1).

### CALCULATING CONE RATIOS

The concentrations of each target (copies/μL) along with metrics of run quality were generated using QuantaSoft Analysis Pro (1.0.596) (Bio‐Rad, US). We calculated the relative abundance of each target by dividing the number of droplets positive for the target opsin gene by the sum of all positive droplets (i.e., L/(S+L+M)). This method uses the total cone population to estimate the relative abundance of each individual cone (Dong et al. 2019). We then calculated the two cone ratios of interest. The L:M ratio for each individual was calculated by dividing the relative abundance of the L cone by that of the M cone. The S ratio was calculated by dividing the relative abundance of the S cone by the summed L and M values. We then averaged the cone ratios across the three triplicate runs for each individual, to calculate a mean that was used for heritability analyses.

### STATISTICAL ANALYSES

We examined variation by calculating the averages, standard errors, and minimum and maximum cone ratios in the sampled populations using R (version 4.0.2 [R Core Team 2019], packages: tidyverse [Wickham et al. 2019], reshape2 [Wickham 2020], lans2r [Polerecky et al. 2012], lubridate [Grolemund and Wickham 2011], and ggplot2 [Wilkinson 2011]). We estimated the narrow‐sense heritability of cone ratios using a quantitative genetic “animal model” (Kruuk 2004), using the MCMCglmm package in R (Hadfield, 2010; Team and Others 2013). For both L:M cone ratio and S:(L+M) cone ratio separately, we fit a mixed linear model that included fixed effects of age, sex, time of sample collection, and year of measure (a two‐level factor distinguishing samples collected in 2018 from 2019 samples). Random effects decompose the phenotypic variance not accounted for by the fixed effects into additive genetic variance and residual (i.e., environmental) variance. We used noninformative Wishart prior distributions for the random effects. Models were run for at least 1.5 million iterations, with a thin interval of 1000 and a burn‐in of 10,000 iterations. All models obtained effective sample sizes of at least 1000, autocorrelations within effects of less than 0.10, and model convergence was checked with visual inspection of density plots. Infants were excluded from analyses, as pedigree information was not yet available for these individuals, resulting in a sample size of 159 individuals between age 3 and age 19 (69 males, 90 females; 70 sampled in 2018, 89 sampled in 2019).

To determine if the additive genetic variance was significant for each cone ratio trait, we compared the deviance information criterion (DIC) between the animal models and the equivalent model excluding the additive genetic variance term (Spiegelhalter et al. 2002). In the case that the model with the additive genetic variance term had a lower DIC value, we determined the additive genetic variance in the cone ratio to be significant. In the case where additive genetic variance was determined to be significant, estimates of the narrow‐sense heritability of the cone ratio trait were calculated as the proportion of total phenotypic variance (additive genetic variance + residual variance) attributed to additive genetic variance.

For each cone ratio trait, we also extracted residuals from the best fit model and tested for significant sex differences in the variance of these residuals using permutation tests (Wierenga et al. 2018; DeCasien et al. 2020; Wierenga et al. 2020). Specifically, we calculated the log male‐to‐female variance across the residuals (positive values = greater male variability; negative values = greater female variability), randomly permuted the sex variable among the residuals 10,000 times, and calculated the proportion of permuted test statistics (absolute value) greater than the observed ratio (absolute value). This proportion is referred to here as “pPERM” and represents a two‐sided test of sex differences in variability.

## Results

### VARIATION AND HERITABILITY OF L:M CONE RATIO

**Figure 2:**
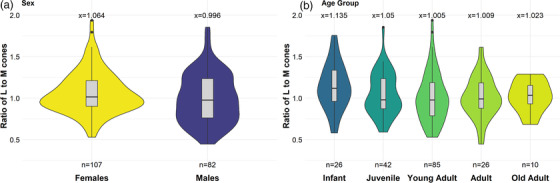
L:M cone ratios of *M. mulatta* by (a) sex and (b) age group. X is the average ratio for each group.

**Figure 3:**
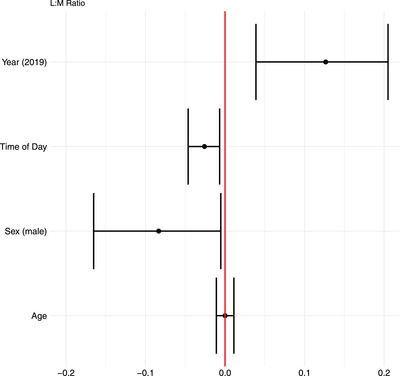
Point estimates and 95% credible intervals of fixed effects from linear models with the lowest DIC for the L:M cone ratio. Effects that span zero (denoted by a red line) are nonsignificant.

The mean L:M ratio (± SE) was 1.03:1 (± 0.0199; range = 0.446–1.938). In the model with the lowest DIC for L:M ratio, there was a marginal effect of sex, with males having smaller L:M ratios (Figs. 2, 3). There was a small effect of time of sample collection, with lower L:M ratios in the afternoon (Figs. 3, S2). The shape of the plot also suggests cone ratio variation among males was higher than among females. However, our analysis of the best fit model residuals suggested that although males do exhibit higher residual variance for this trait (log M/F variance ratio = 0.319 (positive ratio = M > F variance, negative ratio = F > M variance), this difference is not significant (pPERM = 0.32). There was also an effect of the sampling year (Fig. 3), with animals sampled mostly from social group S in 2019 having larger L:M ratios than animals sampled mostly from social group KK in 2018. There was no effect of age on L:M ratios, and the mean of all age groups hovered just above 1 (Table 1). Turning to heritability analyses, the model including a random effect term for additive genetic variance had a higher DIC value (DIC = 310) than the equivalent model excluding this term (DIC = 28.1). This indicates that the model fit was not improved by controlling for additive genetic variance, and thus the contribution of additive genetic effects to the interindividual variation in the trait is negligible. Consequently, interindividual variation in this trait appears to be driven by environmental (i.e., nongenetic) variables.

**Table 1:** Variation in L:M cone ratios of *M. mulatta* by age groups

	Infant	Juvenile	Young Adult	Adult	Old Adult
Age in years	<1	1–4	5–9	10–14	>15
Sample size	26	42	85	26	10
Average ratio	1.135	1.05	1.005	1.009	1.023
Standard error	0.054	0.04	0.032	0.049	0.06
Maximum ratio	1.755	1.854	1.938	1.612	1.289
Minimum ratio	0.581	0.591	0.529	0.446	0.684

### VARIAION AND HERITABILITY OF S:(L+M) CONE RATIO

**Figure 4:**
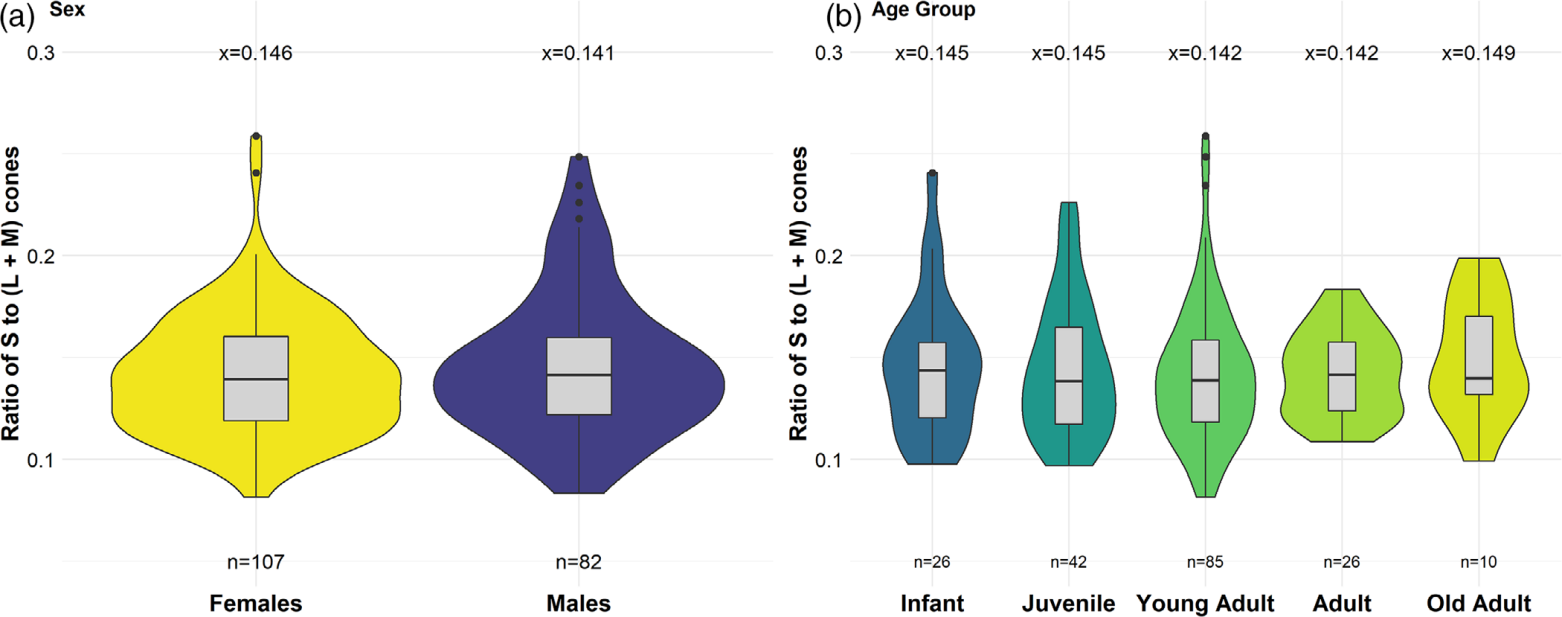
The S: (L+M) cone ratios of *M. mulatta* by (a) sex and (b) age group. X is the average ratio for each group.

**Figure 5:**
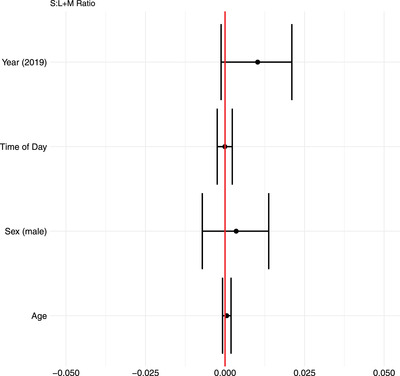
Point estimates and 95% credible intervals of fixed effects from linear models with the lowest DIC for the S: (L+M) cone ratio. Effects that span zero (denoted by a red line) are nonsignificant.

For the S: (L+M) ratio, the mean (± SE) was 0.143 (± 0.002; range = 0.081–0.259) (Table 2). In the model with the lowest DIC, the 95% credible intervals for sex, age, time of day, and year all included zero, indicating no effect of any of these variables on the S cone ratio (Figs. 4, 5). Our analysis of the best fit model residuals suggests that males and females do not differ significantly (log M/F variance ratio = 0.319; pPERM = 0.31). The model that included a random effect term for additive genetic variance had a lower DIC value (DIC = –672.8) than the equivalent model excluding this term (DIC = –647.1), indicating that the additive genetic variance contributes significantly to interindividual variance in the trait. The heritability for S:(L+M) cone ratio was estimated at 43% (CI: 17%–66%).

**Table 2:** Variation in S: (L+M) cone ratios of *M. mulatta* separated by age groups

	Infant	Juvenile	Young Adult	Adult	Old Adult
Age in years	<1	1–4	5–9	10–14	>15
Sample size	26	42	85	26	10
Average ratio	0.145	0.145	0.142	0.142	0.149
Standard error	0.007	0.005	0.004	0.004	0.01
Maximum ratio	0.241	0.226	0.259	0.183	0.199
Minimum ratio	0.098	0.097	0.081	0.109	0.099

## Discussion

We used a gene expression approach to quantify the variation in retinal cone ratios in a large population of free‐ranging macaques. Our main results were fourfold: (1) The average L:M cone ratio hovers near 1:1, that is, an essentially equal ratio of long‐wavelength‐sensitive and mid‐wavelength‐sensitive retinal cones. However, there was considerable variation among individuals, with the L:M ratio ranging from approximately 1:2 to 2:1. (2) We found significant evidence for a mild impact of sex and time of sample collection on L:M ratio, and no evidence for significant additive genetic effects in L:M cone ratio. (3) The ratio of S cones relative to the other (L+M) cone population was on average 0.143 (about 1:7), with less interindividual variation (range = 0.081–0.259). (4) We found no evidence of significant effects of sex, age, or time of sample collection on S:(L:M) cone ratios but do find evidence for significant additive genetic effects in S cone ratio. Below, we discuss the significance of these findings.

### VARIATION AND HERITABILITY OF L:M CONE RATIO

The L:M ratio of rhesus macaques in our study population was 1.03, indicating that these monkeys possess roughly equal numbers of L and M cones on average, with all individuals falling within the range of 0.5–2 for the ratio of L:M cones. This finding is consistent with previous reports of L:M cone equivalency in nonhuman catarrhines based on a microspectrophotometric study of 11 talapoin monkeys (Mollon and Bowmaker 1992), electrophysiological studies of 10 rhesus macaques (Dobkins et al. 2000; Lindbloom‐Brown et al. 2014), ERG studies of 44 rhesus macaques (Jacobs and Deegan 1997), and 26 chimpanzees (Jacobs et al. 1996), as well as gene expression studies of 26 individuals of various catarrhine species (Deeb et al. 2000). Importantly, our large sample size of 189 retinas from unique individuals provides by far the most robust dataset available on nonhuman primate cone ratios and interindividual variation. Our data strongly support previous claims that the 1:1 pattern of catarrhine monkeys deviates strikingly from the human L:M cone ratio, in both the mean ratio, which is roughly 2:1 in humans, and in the range of variation, which can reach 30‐fold in some studies of human cone ratio variation (Carroll et al. 2002; Hofer et al. 2005; Neitz et al. 2006; McMahon et al. 2008). Although the magnitude of interindividual variation of L:M cone ratios was substantially smaller in our study of rhesus macaques when compared to some human studies, the variability in this trait in rhesus macaques was still considerable, ranging fourfold across the study sample. Notably, the additive genetic variance (and consequently the heritability) of L:M cone ratios was negligible in rhesus macaques, suggesting that this variance is primarily driven by environmental and/or nonadditive genetic (e.g., dominance or epistatic genetic) effects. This finding indicates that any selective pressures acting on L:M cone ratios in the population currently are unlikely to result in contemporary evolution of the phenotype in rhesus macaques. This may be one reason why we see such considerable phenotypic variation in the trait persisting in rhesus macaques.

The substantive differences in mean cone ratios between humans and catarrhine monkeys invite discussion of any possible phenotypic outcomes. Maximal chromatic discrimination (i.e., color vision) is achieved when cone types are in similar proportions, which maximizes the chances of detecting chromatic differences present at high spatial frequencies (Gagin et al. 2014). Accordingly, rhesus macaques and the other catarrhine primates examined to date are predicted to have better red‐green hue discrimination than humans. Available behavioral data on the link between cone ratios and chromatic vision support this prediction (Gagin et al. 2014; Horwitz 2015; Gelfand and Horwitz 2018). The adaptive benefits of red‐green color vision have been discussed for decades, and include detection and selection of reddish foods, such as ripe fruit and new leaves, as well as the perception of reddish sociosexual signals (Mollon 1989; Regan et al. 1998; Hiramatsu et al. 2017; Melin et al. 2017; Hogan et al. 2018; Moreira et al. 2019). For example, both male and female rhesus monkeys may be under selection to detect and be attentive to reddish coloration for sociosexual communication (Dubuc et al. 2014; Higham et al. 2021), and this is likely true of other catarrhine monkeys (Setchell et al. 2006; Kamilar et al. 2013), which might explain the narrow range of variation in L:M ratios. Looking time paradigms (Winters et al. 2015) that test behavioral discrimination of colorful stimuli have been undertaken extensively in the present study population (e.g., Higham et al. 2011; Hughes et al. 2015; Dubuc et al. 2016), and hold great promise for testing the links between cone ratios, color discrimination, and spatial acuity.

Conversely, because drawing input from multiple cone types compromises spatial vision at high spatial frequencies—that is, resolution of fine detail and perception of borders and shapes (Bompas et al. 2013; Gagin et al. 2014)—individuals with a greater proportion of one cone type, and individuals with closer cone spectral sensitivities, are predicted to have more acute vision (Danilova et al. 2013; Gagin et al. 2014). Human visual acuity is among the best in the animal kingdom, and is higher than that of macaques and many other catarrhines (Veilleux and Kirk 2014; Caves et al. 2018). A hypothesis of natural selection favoring high visual acuity via cone ratio variation in humans is consistent with other derived features of their visual systems, including eye morphology, and retinal anatomy that supports higher acuity (Veilleux and Kirk 2014; Caves et al. 2018). In support of this, Gagin et al. (2014) found macaques to have improved color vision compared to humans for colors that were modulated by L/M cones. This finding led them to suggest that macaques have higher chromatic sensitivity relatively to humans, at the cost of spatial acuity, although they suggest the effects might be small. Future studies explicitly testing this idea would be fruitful. The genetic mechanism by which humans have doubled their L cone population relative to the M cone population is currently unknown.

The large range in L:M ratio seen among humans (0.22–16.5:1) is additionally surprising (Carroll et al. 2002; Gunther and Dobkins 2002; Hofer et al. 2005), as is variation in other aspects of human color vision, including the high frequencies of recombination between opsin genes, which also differs from other catarrhines (Onishi et al. 1999; Jacobs and Williams 2001; Terao et al. 2005; Hiwatashi et al. 2011). Together, the degradation of human color vision may reflect a diminishing importance of making chromatic discriminations, an increased importance of spatial vision, or both. Links between these features of human vision and other aspects of their evolutionary history, including large cooperative societies and dietary flexibility, remain open and exciting questions.

### VARIATION AND HERITABILITY OF S:(L+M) CONE RATIO

Unlike the L and M cone populations, we find that the S cone ratios of rhesus monkeys are relatively consistent, representing around 14% of the total cone population on average. S cone populations are consistent with those reported in other studies of macaque retinas (Roorda et al. 2001). Unlike with the L:M ratio, we find a tighter range of variation, which might suggest stronger selection on S cone ratios, although it might also reflect the stochasticity of the genetic mechanism of L versus M expression. Interestingly, high levels of variation in S cone ratios are present among some primate species, and linked with activity patterns. For example, some nocturnal primates and other mammals do not have S cones due to pseudogenization of the *OPN1SW* (Szél et al. 1996; Moritz et al. 2013; Melin et al. 2016; Kries et al. 2018). Among nocturnal primates with functional *OPN1SW* opsin genes, S cone ratios range from as little as 0.05% in some mouse lemur species (Dkhissi‐Benyahya et al. 2001; Peichl et al. 2019) to 9%–14% in the spectral tarsier (Hendrickson et al. 2000), and to upward of 13% in other mouse lemur species (Peichl et al. 2019). Among diurnal primates, S cone ratios vary from 3% to as high as 25% in some species (Mollon and Bowmaker 1992; Szél et al. 1996; Bumsted and Hendrickson 1999; Roorda et al. 2001), and constitute about 10% or less of the cone population in humans (Curcio and Hendrickson 1991), but such expression varies from 4% to 15% (Roorda et al. 2001). Our data demonstrate moderate heritability of S cone ratios. In so doing, we provide empirical evidence of the genetic basis by which natural selection may shape the extensive S cone variation present in extant mammals.

### EFFECTS OF AGE AND SEX ON CONE RATIOS

Psychologists studying vision and aging have documented shifts in human color perception with age (e.g., Weinrich et al. 2017). Our results showing that relative cone abundance is consistent across infants, young, middle‐aged, and old macaques rule out developmental remodeling or age‐specific die‐off in some cell types as a significant factor shaping age‐related perceptual shifts. This result is consistent with literature on retinal development and photoreceptor persistence in humans and nonhuman primates (Aboelnour et al. 2017; Weinrich et al. 2017; Kam et al. 2019). We also find that the L:M cone ratio of samples collected later in the day was slightly lower. The biological importance of this is uncertain, and this pattern differs from the large diel variation seen in some vertebrates, such as teleost fish (Halstenberg et al. 2005). Whether macaques in our study population experience changes in color perception due to other forces, such as lenticular senescence (Mellerio 1987; Pokorny et al. 1987), including the yellowing of the lens due to the natural absorption of blue light with age (Pokorny et al. 1987; Lutze and Bresnick 1991), or reduced photoreceptor function (Wang et al. 2010; Aboelnour et al. 2017), remains an open question for future research.

We found a small impact of sex on the cone mosaic patterning, with males showing a bias toward M‐sensitive cones. Differences in retinal mosaics have been previously reported for sexually dimorphic species of birds and reptiles (Bloch 2015; Tseng et al. 2018). For example, in green‐spotted grass lizards, significant differences of opsin expression between the sexes occur during the breeding season, which may permit females to better discriminate among potential mates (Tseng et al. 2018). Similarly, in some warblers, species with plumage dichromatism also have differences in opsin expression between sexes, suggesting mate choice is not only affecting coloration in males, but also visual abilities in females (Kokko et al. 2003; Bloch 2015). However, the sex differences were much greater in these studies than we detect among macaques. Rather, the overarching similarity between males and female macaque retinal mosaics is consistent with studies of other mammalian retinas (Bowmaker 1998; Temple 2011; Viets et al. 2016; Baden and Osorio 2019). One notable exception is the sex difference in the retinas of primates in the Americas, where X‐linked allelic polymorphism of the *OPN1LW* gene leads to male retinas having two cone types and retinas of heterozygous females possessing three cones types (Jacobs 1998; Kawamura et al. 2012), with strong perceptual implications (Caine 2002; Saito et al. 2005). We do not know if the marginal differences between sexes observed in rhesus macaques lead to perceptual differences, but this seems unlikely given the small magnitude of variation. Rather, we find evidence of excellent color perception overall in both sexes, which may reflect strong selective pressures on the L:M cone ratio to facilitate perception of the reddish social signals used by both males and females in this species (Higham et al. 2021).

Differences in gene expression between sexes are not uncommon, and differences in expression of autosomal genes have been reported in a variety of mammals (Naqvi et al. 2019; Oliva et al. 2020; Hägg and Jylhävä 2021). In general, modulation of expression of X‐linked genes is accomplished by upregulation of X‐genes in single‐X individuals, or by random X‐inactivation during embryogenesis in multi‐X individuals (Disteche 2016; Raznahan and Disteche 2021). However, genes do escape these measures, which results in higher expression levels of X‐linked genes in individuals with more X‐chromosomes (Disteche 2016). X‐inactivation genes have been reported to contribute to eye diseases (Suzuki et al. 2019; Mirzaei et al. 2020; Simcoe et al. 2020). For example, in humans and rats, the gene *GPM6B* may escape X‐inactivation and result in higher intraocular pressure, which could lead to glaucoma (Mirzaei et al. 2020; Simcoe et al. 2020). Additional studies are improving our understanding of differential sex bias expression in mammals (Bwire, 2020; Ytrehus et al. 2021), but more research is needed on the extent of sex‐biased gene expression in different tissues, how these differences in expression affect phenotypes, and how phylogeny can drive these changes (Naqvi et al. 2019; Wilson 2021). Our study raises an intriguing possibility of sex impacts on cone ratio variation, but more research is needed to robustly assess this.

## Summary

The evolution of uniform trichromacy via a gene duplication is one of the foundational features of catarrhine evolution. It is thought to have opened up new dietary niches in the detection of colorful fruits, flowers, and young leaves against green backgrounds (Dominy and Lucas 2001; Melin et al. 2009; Carvalho et al. 2017), and enabled selection on colorful sociosexual signals (Hiramatsu et al. 2017; Higham et al. 2021), making primates the most colorful order of mammals (Bradley and Mundy 2008; Caro et al. 2021). Our research uses a large sample size (*n* = 189) to explore cone ratio variation via opsin gene expression in a nonhuman primate model and is the first to examine the genetic and environmental contributors to interindividual variation. We provide clear evidence that rhesus macaques have 1:1 L:M cone ratios, with little interindividual variation, a pattern that is similar to other nonhuman catarrhines but clearly differs from the pattern seen in humans. We further provide evidence that S but not L:M cone ratios are heritable, leaving the derived 2:1 (L:M) ratio seen in humans a puzzle for future research. Together this research provides insight into the evolutionary pressures shaping color vision and other visual features in our lineage.

## AUTHOR CONTRIBUTIONS

RAM, JPH, and ADM contributed equally to the writing and organization of the manuscript. RAM and ADM designed the molecular analyses. JPH and ADM conceived of the study, with input from SK. Data collection was done by RAM, MCJ, ARD, SBS, and CBRU. Genetic extractions, ddPCR, and analyses were done by RAM and LGL. Heritability analyses were performed by EBC with help from ADM. Field project oversight and access to primate samples were facilitated by JPH, MJM, and MIM. All authors were involved with reviewing and editing the manuscript.

## CONFLICT OF INTEREST

The authors declare no conflict of interest.

## DATA ARCHIVING

The data and code underlying this article are available via DRYAD (https://doi.org/10.5061/dryad.gf1vhhmsc).

Associate Editor: Dr. Juan Diego Gaitan‐Espitia

Handling Editor: Prof. Tracey Chapman

## Supporting information



Supplementary Table 1: Caribbean Primate Research Unit Stakeholders (alphabetical order).
Supplementary Table 2: Primers and probes, with melting temperature (Tm), and primer/probe concentrations used for *OPN1SW*, *OPN1MW*, & *OPN1LW* opsin genes of *Macaca mulatta*.
Supplementary Table 3: L:M cone ratio‐testing ddPCR results from synthetic cDNA of L and M opsins.
Supplementary Table 4: Thermal cycle settings for amplification.
Supplementary Figure 1: Visualization of the duplex assays generated using ddPCR, demonstrating controlled ratio‐testing between L and M opsins. The L:M ratio is noted in the upper left‐hand corner of each panel.
Supplementary Figure 2: Ratio of the L:M cones by time of sample collection. Regression lines are separated by sex.

SUPPORTING INFORMATION
