# Supplementary material for: Variation and heritability of retinal cone ratios in a free‐ranging population of rhesus macaques
Source: Evolution. 2022 Jul 19;76(8):1776–89. doi: 10.1111/evo.14552 (PMC9544366; doi:10.1111/evo.14552)
Supplement: Supplementary file 1 — Supplementary Table 1: Caribbean Primate Research Unit Stakeholders (alphabetical order). Supplementary Table 2: Primers and probes, with melting temperature (Tm), and primer/probe concentrations used for OPN1SW, OPN1MW, & OPN1LW opsin genes of Macaca mulatta. Supplementary Table 3: L:M cone ratio‐testing ddPCR results from synthetic cDNA of L and M opsins. Supplementary Table 4: Thermal cycle settings for amplification. Supplementary Figure 1: Visualization of the duplex assays generated using ddPCR, demonstrating controlled ratio‐testing between L and M opsins. The L:M ratio is noted in the upper left‐hand corner of each panel. Supplementary Figure 2: Ratio of the L:M cones by time of sample collection. Regression lines are separated by sex. [file EVO-76-1776-s001.pdf]

SUPPORTING INFORMATION FOR:

Variation and heritability of retinal cone ratios in a free-ranging population of rhesus macaques

Rachel A. Munds, Eve B. Cooper, Mareike C. Janiak, Linh Gia Lam, Alex R. DeCasien, Samuel Bauman Surratt, Michael J. Montague, Melween I. Martinez, Cayo Biobank Research Unit, Shoji Kawamura, James P. Higham, Amanda D. Melin

SUPPLEMENTARY TABLES:

Supplementary Table 1: Caribbean Primate Research Unit Stakeholders (alphabetical order).

Stakeholder	Affiliations
Lauren J.N. Brent	Centre for Research in Animal Behavior, University of Exeter, Exeter, UK
James P. Higham	Department of Anthropology, New York University, New York, NY, USA
Melween I. Martinez	Caribbean Primate Research Center, University of Puerto Rico, San Juan, PR, USA
Amanda D. Melin	Department of Anthropology & Archaeology, University of Calgary, Calgary, AB, CAN<br>Department of Medical Genetics, University of Calgary, Calgary, AB, CAN<br>Alberta Children's Hospital Research Institute, University of Calgary, Calgary, AB, CAN
Michael J. Montague	Department of Neuroscience, University of Pennsylvania, Philadelphia, PA, USA
Michael L. Platt	Department of Neuroscience, University of Pennsylvania, Philadelphia, PA, USA<br>Department of Psychology, University of Pennsylvania, Philadelphia, PA, USA<br>Department of Marketing, University of Pennsylvania, Philadelphia, PA, USA
Noah Synder-Mackler	Center for Evolution & Medicine, Arizona State University, Tempe, AZ, USA<br>School of Life Sciences, Arizona State University, Tempe, AZ, USA

Supplementary Table 2: Primers and probes, with melting temperature (Tm), and primer/probe concentrations used for *OPN1SW*, *OPN1MW*, & *OPN1LW* opsin genes of *Macaca mulatta*.

Region	Name	Sequence (5'-3')	Tm	Concentration
**Primers**				
OPN1SW	S_F	CTCTGTCTTCCCTGTCTTTGTC	62	1350nm
	S_R	TCACCAGACCTGCTACAGT	62	
OPN1MW & OPN1LW	Ex5F (778-799)	CAGAAGGCAGAGAAGGAAGTG	62	900nm
	Ex5R (855-875)	CAGCAAAGCATGCGAAGAAG	62	
**Probes**				
OPN1SW	S_opsin	TTGGTCGCCATGTTTGTGCTTTCG	68	375nm
OPN1LW	L (818-842)	TGATCTTCGCATACTGCGTCTGCT	68	250nm
OPN1MW	M (818-842)	TGTTCCTGGCGTTCTGCTTCTGCT	70	250nm

Supplementary Table 3: L:M cone ratio-testing ddPCR results from synthetic cDNA of L and M opsins.

Ratio	L Concentration (copies/µL)	M Concentration (copies/µL)
15:1	121.37	7.37
4:1	97.10	28.56
2:1	89.77	39.85
1:1	63.84	65.95
1:2	42.51	86.15
1:4	26.25	93.03
1:15	7.70	107.51

Supplementary Table 4: Thermal cycle settings for amplification.

Step	Temperature (C)	Time (minutes)	Ramp	Number of cycles
Enzyme Activation	95	10	1C/sec	1
Denaturation	94	0.5	1C/sec	50
Annealing	57	2	1C/sec	50
Enzyme Deactivation	98	10	1C/sec	1
Chill	4	10	1C/sec	1
Hold	4	Infinite	1C/sec	1

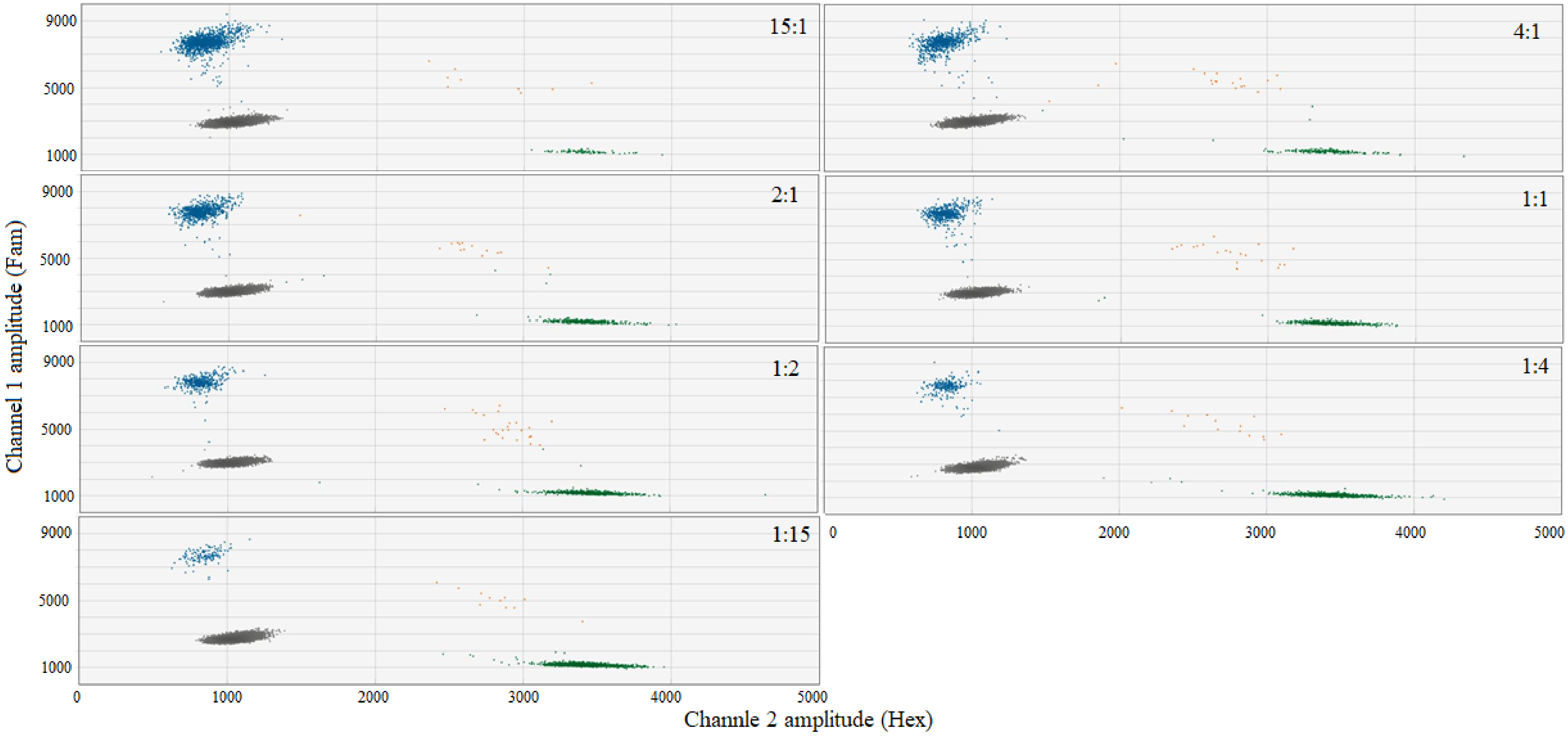

Supplementary Figure 1: Visualization of the duplex assays generated using ddPCR, demonstrating controlled ratio-testing between L and M opsins. The L:M ratio is noted in the upper left-hand corner of each panel.

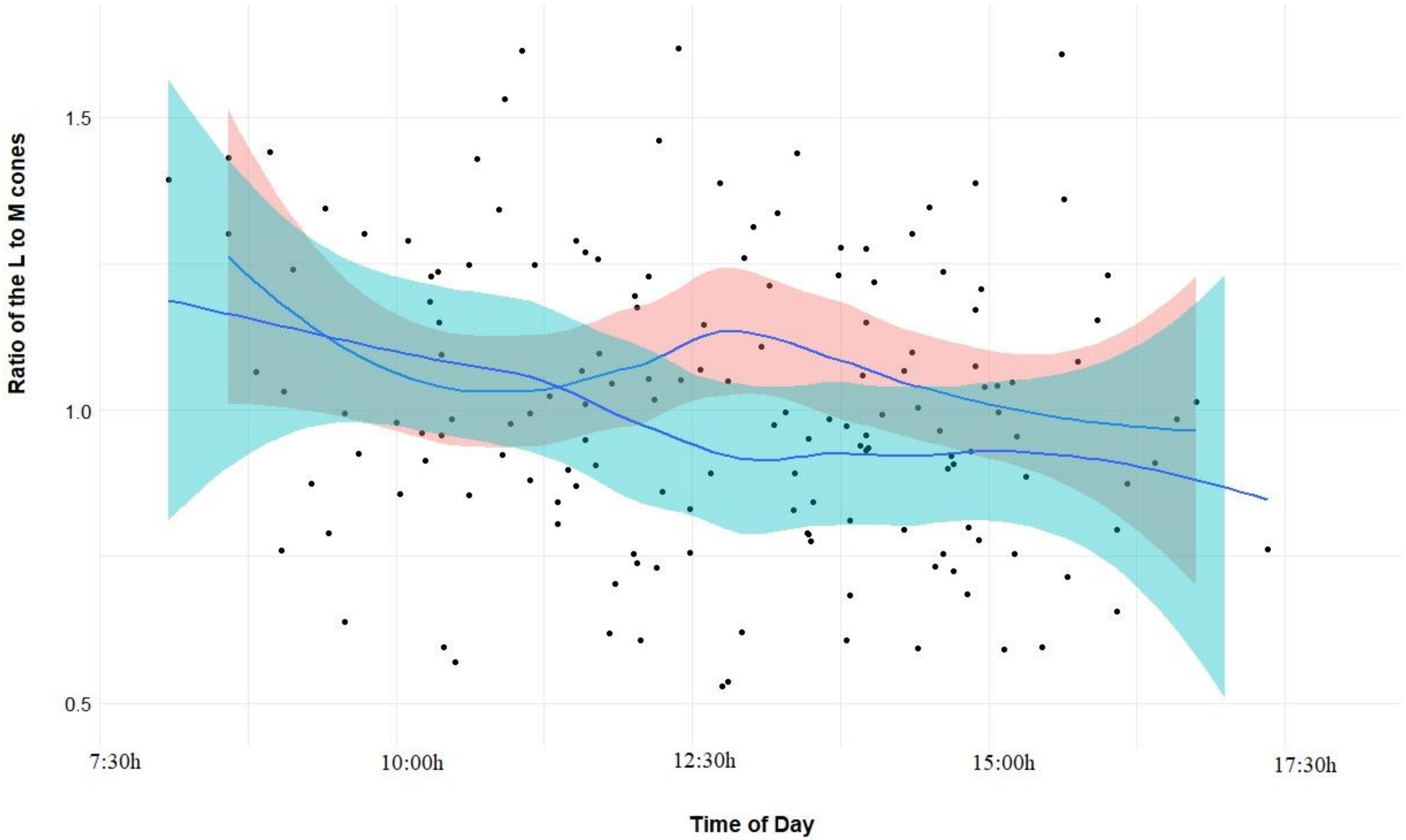

Supplementary Figure 2: Ratio of the L:M cones by time of sample collection. Regression lines are separated by sex.
