# Supplementary material for: Variation and heritability of retinal cone ratios in a free‐ranging population of rhesus macaques
Source: Evolution. 2022 Jul 19;76(8):1776–89. doi: 10.1111/evo.14552 (PMC9544366; doi:10.1111/evo.14552)
Supplement: Supplementary file 2 — SUPPORTING INFORMATION [file EVO-76-1776-s002.pdf]

RNA isolation using TRIzol and Qiagen RNeasy kit from retinal tissue

PROCEDURE

1. Either keep tissue in 1 ml TRIzol, or add 1 ml TRIzol to a small tissue biopsy.
2. Homogenize with 26/27gauge needle using a 1ml or 3ml syringe until tissue is completely homogenized (~20 pumps). Keep the samples in the mini-cooler when not homogenizing.
    a. During homogenization, you will transfer the sample to a 1.5ml tube using a 1000ul pipette. After transfer, use the 1000 pipette and pump 3-5 times to start tissue homogenization before using the syringe & needle.
    b. Do not use the same pipette or needle/syringe for different samples to avoid contamination.
3. Add 200 ul chloroform per ml of TRIzol. **Vortex 15 seconds** and **incubate at room temperature for 3 minutes.**
4. Centrifuge at **4 C** for 15 minutes at maximum speed
    a. After this step the centrifuge can be warmed to 23-25C.
5. Carefully transfer 400 ul of the clear, colourless supernatant to a clean sterile 1.5 ml tube. **Avoid the middle, white layer**. Dispose of the remaining liquid in the liquid waste.
    - Three phases will form, a pink TRIzol layer on the bottom, a white DNA inter-phase layer in the middle, and a top colorless RNA layer.
6. To the 1.5 ml tube, add an equal volume (400 ul) of 70% ethanol to the collected supernatant, mix well through pipetting.
7. Transfer 700 ul to an RNeasy spin column placed in a 2mL collection tube. Centrifuge at room temperature for 1 minute at 10 000 rpm. Discard the flow-through and transfer any remaining solution to the column and centrifuge again. Transfer the flow through column to a new collection tube.
8. Wash with 350 ul Buffer RW1. Centrifuge at room temperature for 1 minute at 10 000 rpm. Discard the flow through with a pipette.
9. In a separate tube, add 10 uL DNase I Stock Solution to 70 uL Buffer RDD. Mix by gently inverting the tube, and centrifuge briefly to collect residual liquid from the sides of the tube.
10. Carefully add the 80 uL mix from step 9 directly to the RNeasy spin column membrane (from step 8), and place on the benchtop (20–30°C) for 15 min.
11. Add 350 µl Buffer RW1 to the RNeasy spin column. Close the lid gently, and centrifuge for 1 minute at 10 000 rpm. Discard the flow through with a pipette.
12. Wash with 500 ul Buffer RPE. Centrifuge at room temperature for 1 minute at 10 000 rpm. Discard the flow through with a pipette.
13. Repeat step 12: wash with 500 ul Buffer RPE. Centrifuge at room temperature for 1 minute at 10 000 rpm. Discard the flow through with a pipette.
14. Centrifuge at room temperature for 2 minutes at 13 000 rpm. This step removes any ethanol or other washes. Do not skip it!
15. Transfer the column to a clean, sterile 1.5 ml Eppendorf tube. Elute RNA with 40 ul RNase free water. Incubate at room temperature for 5 minutes. Centrifuge at room temperature for 1 minute at 13000 rpm.
16. Repeat step 15 for a second elution of RNA, except extend incubation to 10 minutes.
17. Immediately Qubit the extracted RNA or store at -20 or -80 until further analyses.
